# Identification of the Shared Gene Signatures between Autism Spectrum Disorder and Epilepsy via Bioinformatic Analysis

**DOI:** 10.1155/2022/9883537

**Published:** 2022-12-16

**Authors:** Yuexia Xu, Yifeng Wang, Baomei He, Yuhui Yao, Qianqian Cai, Lihui Wu

**Affiliations:** ^1^Department of Children's Health Care, The Second Affiliated Hospital & Yuying Children's Hospital, Wenzhou Medical University, 109 West Xueyuan Road, Wenzhou 325003, China; ^2^Department of Clinical Medicine, Hangzhou Medical College, Hangzhou, China; ^3^School of Medicine & Health Science, The George Washington University, Ross Hall, 2300 Eye Street, NW, Washington DC 20037, USA; ^4^Center for Reproductive Medicine, Department of Pediatrics, Zhejiang Provincial People's Hospital (Affiliated People's Hospital, Hangzhou Medical College), Hangzhou, Zhejiang, China 310014; ^5^Shanghai Key Laboratory of Molecular Imaging, Shanghai University of Medicine and Health Sciences, Shanghai 201318, China

## Abstract

**Purpose:**

To identify gene signatures that are shared by autism spectrum disorder (ASD) and epilepsy (EP) and explore the potential molecular mechanism of the two diseases using WGCNA analysis. Additionally, to verify the effects of the shared molecular mechanism on ADHD, which is another neurological comorbidity.

**Methods:**

We screened the crosstalk genes between ASD and EP based on WGCNA and differential expression analysis from GEO and DisGeNET database and analyzed the function of the genes' enrichment by GO and KEGG analyses. Then, with combination of multiple datasets and multiple bioinformatic analysis methods, the shared gene signatures were identified. Moreover, we explored whether the shared gene signature had influence on the other neurological disorder like ADHD by analyzing the difference of the relative genes' expression based on bioinformatic analysis and molecular experiment.

**Results:**

By comprehensive bioinformatic analysis for multiple datasets, we found that abnormal immune response and abnormal lipid metabolic pathway played important roles in coincidence of ASD and EP. Base on the results of WGCNA, we got the hub genes in ASD and EP. In attention deficit and hyperactivity disorder (ADHD) animal model, we also found a significant difference of gene expression related to sulfatide metabolism, indicating that the abnormal sphingolipid metabolism was common in multiple neurological disorders.

**Conclusion:**

This study reveals shared gene signatures between ASD and EP and identifies abnormal sphingolipid metabolism as an important participant in the development of ASD, EP, and ADHD.

## 1. Introduction

The comorbidity among neurological diseases has attracted a significant amount of attention because its complex symptoms have brought great challenges to diagnosis and treatment in recent years [1]. However, there is still a limited understanding of the etiology and pathogenesis of the comorbidity among the neurological diseases. The co-occurrence of these diseases highly suggests a common neurophysiological mechanism between them. Autism spectrum disorder (ASD) and epilepsy (EP) are common neurological diseases that have early onsets in childhood. Epidemiological study has pointed out that the prevalence of ASD in patients with EP is 6.3%, which is much higher than that in the general population (0.75%-1.1%) [2]. The prevalence of EP for ASD patients can reach to 25% [3]. These statistics constantly remind us of the frequent comorbidity between the two diseases, which also suggests that both disorders may be introduced by the same pathogenesis. It has been acknowledged that the genetics play an important role in the pathogenesis of ASD [4] and the EP [5], indicating that both disorders may have common genetic basis. However, the common genetic basis has not still been revealed clearly in previous studies, which only provided hypotheses suggesting the imbalance between excitation and inhibition state of the brain, abnormal synaptic plasticity, and gene transcriptional regulation may cause the occurrence of ASD and EP [6–10]. It is also reported that synapse-related gene mutations exited in patients with ASD and EP, and synapse is the key link of neuronal excitation and inhibition [11–13]. In addition, research integrating a large number of EP- and ASD-associated genes in the multiplex network obtained some shared genes that are enriched in ion transmembrane transport and synaptic signaling [14]. Weighted gene coexpression network analysis (WGCNA) [15] is a systems biology method used to describe the gene association patterns between different samples and can possibly identify highly synergistic gene series. WGCNA can identify the relationship among a large amount of gene information and convert them into the relationship between gene set with similar functional structure and phenotype, avoiding multiple hypothesis test and correction issue. To determine the genetic link between ASD and EP furtherly, we used WGCNA analysis to screen overlapping genes with dysregulated expression in ASD and EP, namely, crosstalk genes, to explore the potential molecular mechanism of the comorbidity. This study is aimed at exploring the comorbidity between ASD and EP by identifying the shared genetic characteristics and molecular mechanisms between these two diseases based on WGCNA.

## 2. Method

### 2.1. Extraction of Microarray Data about ASD and EP

**Figure 1:**
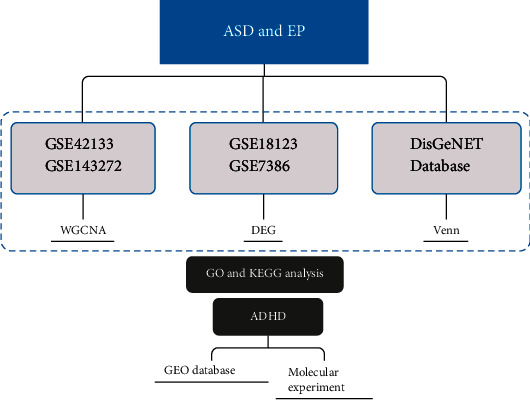
The flow diagram.

The gene expression profile of autism spectrum disorder and epilepsy was downloaded from the Gene Expression Omnibus (GEO) of NCBI (http://www.ncbi.nlm.nih.gov/gds/) by using the keywords “autistic spectrum disorder” or “ASD” and “epilepsy”. In our study, the screening criteria of many datasets were as follows: first, the samples in the dataset must include both case group and control group; second, all of the data samples explored in this study should be consistent and collected from blood samples; third, the dataset must provide preprocessed data or original data that can be used for reanalysis; fourth, the number of samples in each group shall not be less than 10 to ensure the accuracy of WGCNA analysis results; fifth, all of the dataset samples should be collected from humans. Based on such screening criteria, two datasets related to ASD (GSE18123 and GSE42133) and two datasets related to EP (GSE7486 and GSE143272) were included in this study. The detailed information of the dataset is shown on Table 1, and the detailed flow diagram is shown in Figure 1.

### 2.2. Identifying the Significant Modules in ASD and EP by WGCNA

GSE42133 and GSE143272 from the GEO database were selected as the analysis objects, and all genes of the datasets were annotated and standardized, respectively. And then, the gene expression values were transformed into the matrixes. A total of the top 5000 genes were screened which have the greatest difference in expression for further analysis based on the “WGCNA” package (version 1.70-3) of R program (version 4.0.5) to obtain the significant modules. More specifically, the gene matrixes were converted to adjacency matrixes by using Pearson's correlation coefficients and established the unsupervised coexpression relationship. The sample clustering tree was constructed by the “Hclust” function to remove outlier samples, and the power *β* was calculated by the “pickSoftThreshold” function to make *R*^2^ greater than 0.8, so that the constructed gene network basically conformed to the scale-free topology criterion. Based on the adjacency matrix, the topological overlap (TOM) matrix and dissimilarity matrix were established. Next, the genes were matched with the modules of different colors by dynamic tree cut, and the number of the genes in a module is generally no less than 30. Other genes that did not match the module were uniformly divided into gray modules. Finally, the correlation between the modules and the clinical feature was calculated to identify significant modules about ASD and EP separately.

### 2.3. Identifying the Crosstalk Genes between ASD and EP

The significant modules related to ASD and EP were screened, and the related genes were extracted. The intersection between ASD- and EP-related genes was organized by Venn diagram. This intersection is the crosstalk gene between ASD and EP. Functional analysis was performed to identify the biological functions of crosstalk genes, especially from the aspects of biological processes and signal pathways.

### 2.4. Shared Gene Signatures between ASD and EP Based on DisGeNET Database

DisGeNET (http://www.disgenet.org) is a database specially containing the information of genes and mutation sites related to human diseases. The genes related to ASD and EP were extracted, respectively, and the Gene Ontology (GO) and Kyoto Encyclopedia of Genes and Genomes (KEGG) were performed to get the shared gene signatures between ASD and EP.

### 2.5. Differential Expression Analysis

Differential expression analysis was performed for the GSE7486 and GSE18123 dataset using the “limma” package in R program. The genes with a *P*value < 0.05 and ∣log FC | ≥0.5 were regarded as differentially expressed genes (DEGs). We used Venn diagram to obtain the crosstalk genes between ASD and EP among DEGs, and we got the shared gene signatures between ASD and EP from function analysis of the crosstalk genes.

### 2.6. Analysis of Imbalance of Sphingolipid Metabolism Based on Database

The datasets were downloaded from GEO database including GSE1675, GSE2116, GSE8051, GSE8796, GSE12457, GSE41552, GSE53363, and GSE144548 to observe the expression difference of target gene between SHRs and WKYs.

### 2.7. Analysis of Imbalance of Sphingolipid Metabolism Based on Molecular Biology Experiment

Five-week-old male spontaneously hypertensive rats (SHR) and Wistar Kyoto (WKY) rats purchased from the Charles River Laboratories (Beijing, China) were divided into 2 groups, respectively, in this study, and there were 5 rats in each group. Before the experiment, all the rats were adaptively fed for one week, with free drinking and eating. When the rats were six-week-old, ethological experiments including the open field test (OFT) and the Làt maze were performed to evaluate difference of behaviors between two groups. In the OFT, we put a box which was 90 cm × 90 cm × 50 cm cube on the ground, with the black inner wall and the bottom divided into 9 squares. Then, a camera was set up above the box about 2 m to ensure that its field of vision can cover the whole inside of the box. When the rat placed on the corner grid facing the wall was allowed to roam free for 5 minutes, we recorded their activities including the number of square crossings and the number of rearing by the camera to observe the horizontal and vertical activity levels of rats. In the Làt maze, a 30 cm × 30 cm × 40 cm plexiglass box was placed in the middle of a 60 cm × 60 cm × 40 cm box to make the rats move between the big box and the glass box. We also fixed a camera above the equipment which was used to record the rats' free activities for 30 minutes. We counted the number of square crossings and the number of rearing, which represented the general activity level and nonselective attention level of rats. We obtained the prefrontal cortex (PFC) and hippocampus (Hip) of the rats. Moreover, the total RNA was extracted from PFC and Hip with TRIzol reagent (Invitrogen, USA) according to the manufacturer's instructions and was reverse transcribed to cDNA, which was conducted as a template to perform polymerase chain reaction (PCR) and real-time polymerase chain reaction (QPCR). The specific primers used in the experiment are listed on Table 2.

## 3. Result

### 3.1. Identifying Significant Modules and Genes in ASD and EP by WGCNA

**Figure 2:**
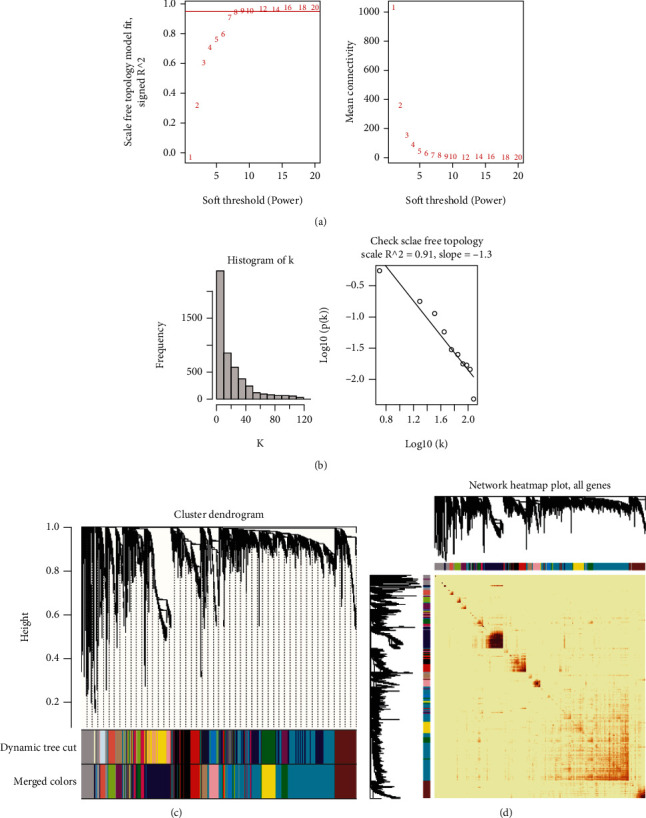
Weighted gene coexpression network analysis (WGCNA) of GSE42133. (a) Scale independence and mean connectivity. (b) The scale-free topology when *β* = 7. (c) The cluster dendrogram of coexpression genes in ASD. (d) Network heat map of all genes.

**Figure 3:**
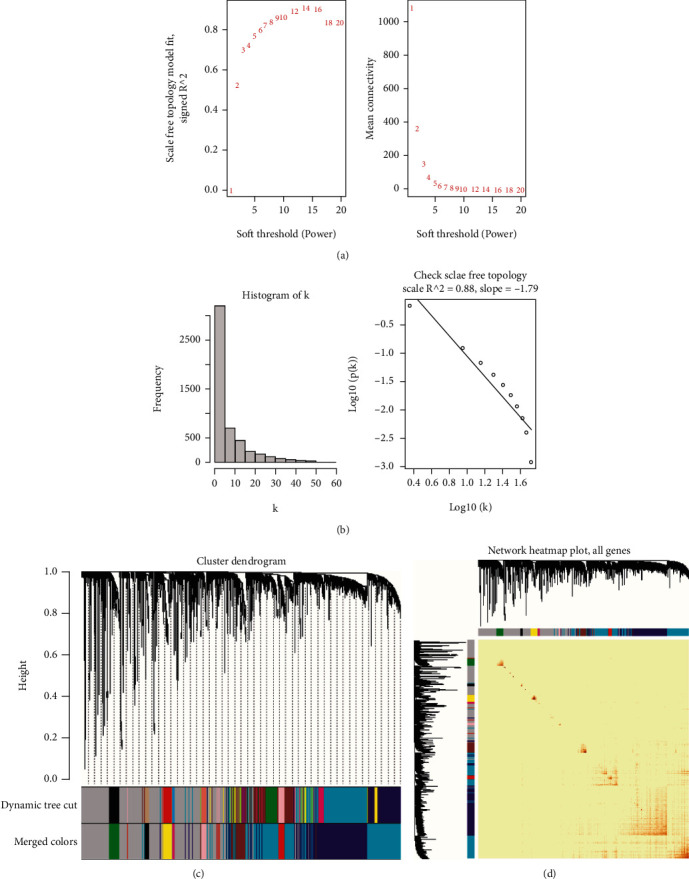
Weighted gene coexpression network analysis (WGCNA) of GSE143272. (a) Scale independence and mean connectivity. (b) The scale-free topology when *β* = 9. (c) The cluster dendrogram of coexpression genes in EP. (d) Network heat map of all genes.

**Figure 4:**
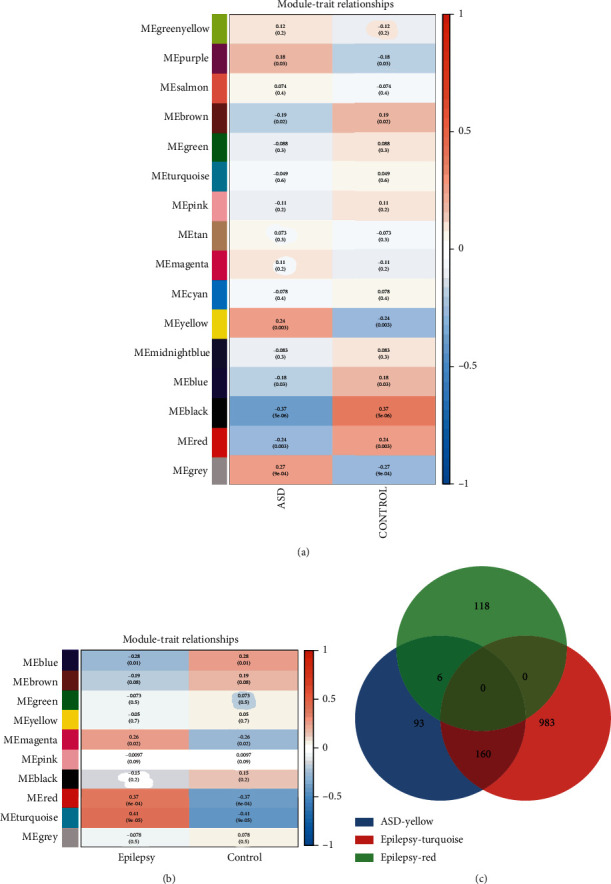
Identifying significant modules of ASD and EP. (a) Module–trait relationships in ASD. Each cell contains the corresponding correlation and *P* value. (b) Module–trait relationships in EP. (c) Crosstalk genes between ASD and EP.

We firstly analyzed the sample data consisting of the 91 ASD patients and 56 healthy individuals from GSE42133. The soft-threshold power (*β*) was calculated as 7 according to the scale independence and mean connectivity (Figure 2(a)), and we found that the scale-free topology was good when *β* was 7 because *R*^2^ was greater than 0.85 (Figure 2(b)), suggesting biological significance. As it is shown in Figures 2(c) and 2(d) that all genes were classified into different modules in GSE42133. Similarly, GSE143272 dataset was analyzed by using the same analysis method, and the results were displayed in Figure 3 when *β* was determined to be 9. It was particularly noteworthy that we only selected 85 samples that were not affected by drugs, which consisted of 51 healthy individuals and 34 EP patients from GSE143272. Given the analysis of GSE143272, the scale-free topology was good when *β* was 9 because *R*^2^ was 0.88 (Figures 3(a) and 3(b)), and the cluster dendrogram of coexpression genes and network heat map was displayed in Figures 3(c) and 3(d). A total of 16 modules were identified in GSE42133 according to the WGCNA (Figure 4(a)). Genes in GSE143272 were divided into 10 modules (Figure 4(b)). In the analysis of ASD dataset, yellow module (*r* = 0.24, *P* = 0.003), black module (*r* = −0.37, *P* = 5*e* − 06), and red module (*r* = −0.24, *P* = 0.003) were positively correlated with ASD phenotype. In the analysis of EP dataset, red module (*r* = 0.37, *P* = 6*e* − 04) and turquoise module (*r* = 0.41, *P* = 9*e* − 05) were positively correlated with EP phenotype. We extracted the genes in these modules and did intersection analysis by Venn diagram, to find 166 overlapping genes (Figure 4(c)).

### 3.2. Function Analysis of Crosstalk Genes from the Result of WGCNA

**Figure 5:**
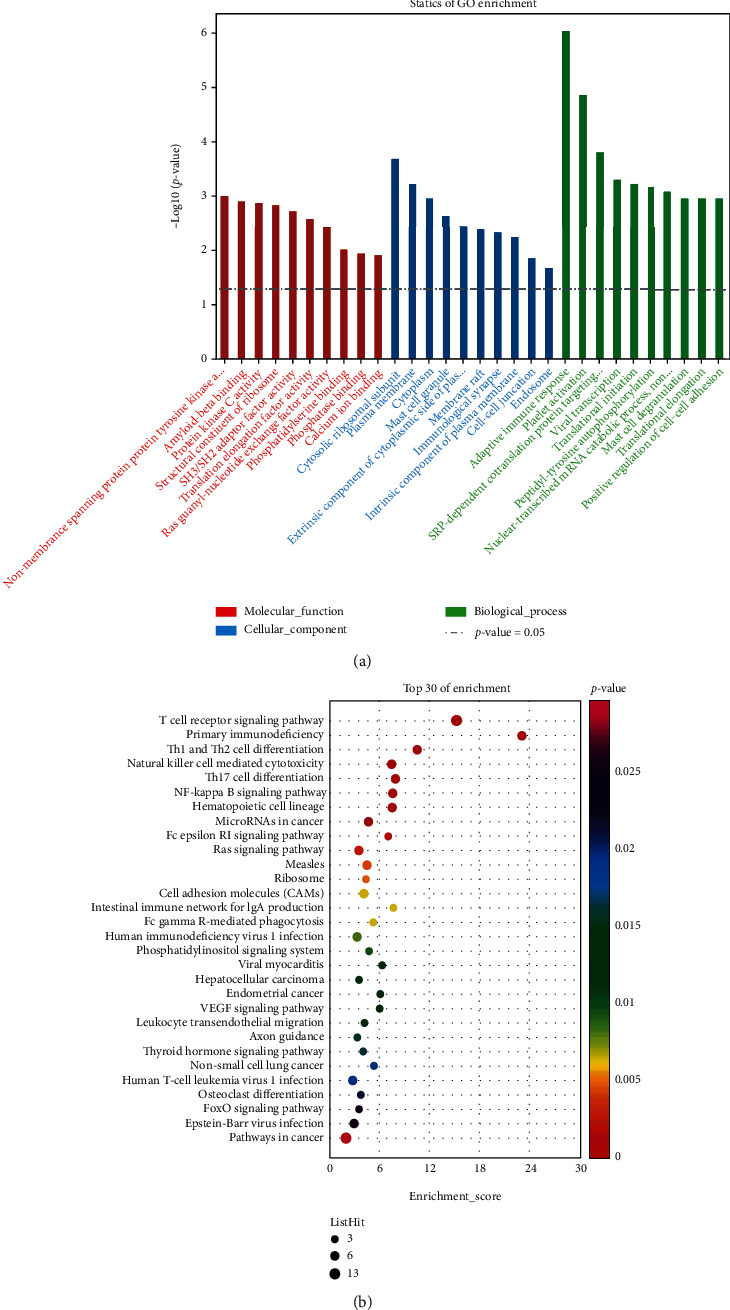
The enrichment analysis of 166 crosstalk genes. (a) Gene Ontology (GO). (b) Kyoto Encyclopedia of Genes and Genomes (KEGG).

**Figure 6:**
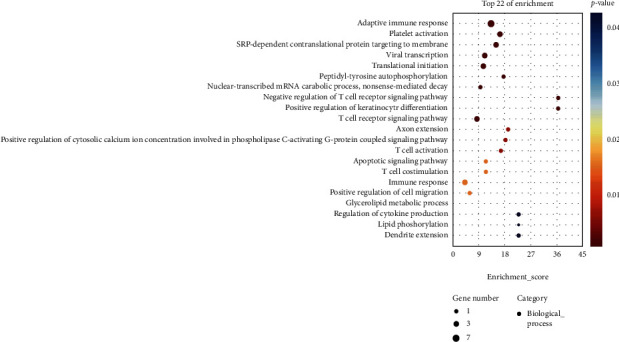
The biological process analysis of the crosstalk genes.

A total of 166 crosstalk genes between ASD and EP were filtered by WGCNA. Using analysis of GO and KEGG, we found that the crosstalk genes were mostly associated with immune pathways. As Figure 5 showed, for biological process, most crosstalk genes were enriched in adaptive immune response, followed by platelet activation. From the analysis of KEGG, we got the top 30 of enrichment pathways, in which the top 5 enriched pathways were related to immune system, especially T cell immune pathways. Through further analysis of the 166 genes, we also discovered that the lipid metabolism was involved in these diseases in addition to the immune response. As shown in Figure 6, the *P* values of glycerolipid metabolic process and lipid phosphorylation are significant (0.027 and 0.042) according to the GO analysis of the crosstalk genes based on WGCNA.

### 3.3. Analysis of Genes between Related to ASD and EP from the DisGeNET Database

**Figure 7:**
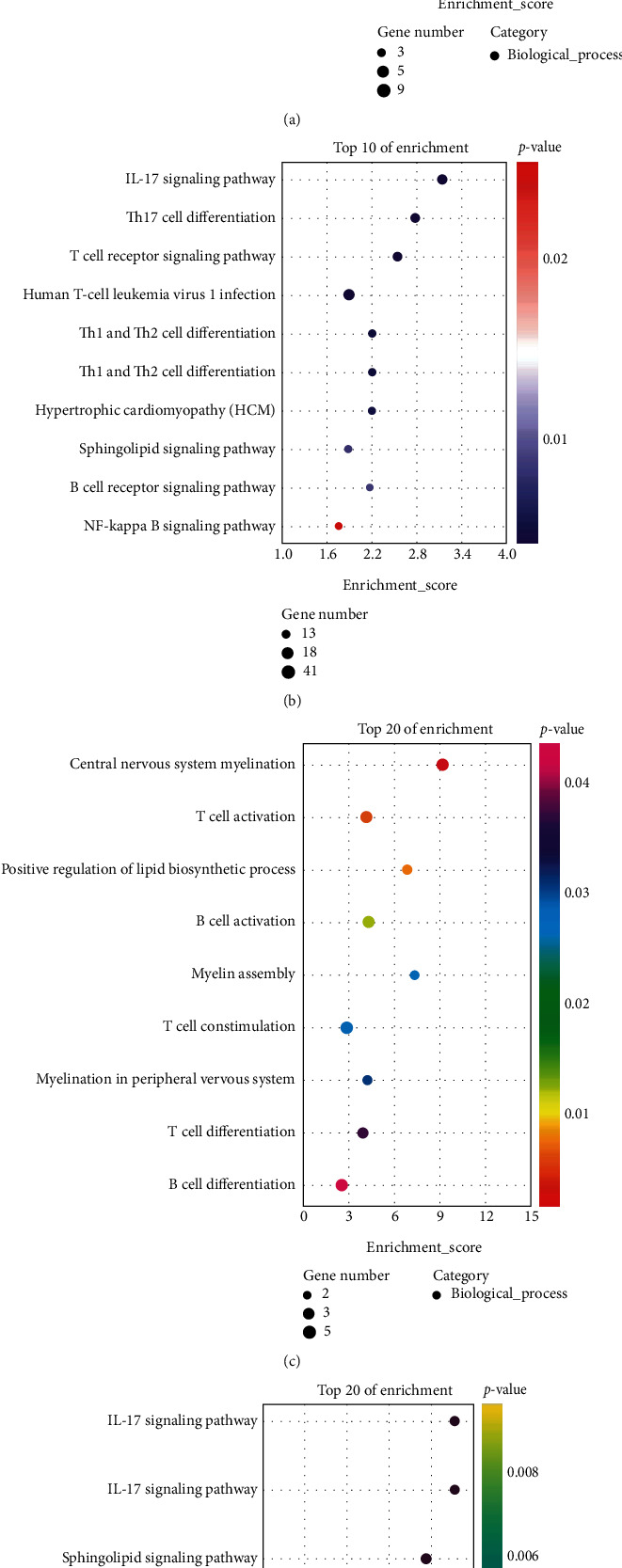
The functions regulated by genes related ASD and EP from DisGeNET Database. (a) The biological process of gene enrichment related to ASD. (b) KEGG analysis of genes related to ASD. (c) The biological process of gene enrichment related to EP. (d) KEGG analysis of genes related to EP.

We extracted 1071 genes related to ASD (c1510586) and 1215 genes related to EP (c0014544) from the DisGeNET database and then performed GO analysis and KEGG analysis on these genes, respectively. We extracted the GO terms where the *P*value < 0.05 from the biological process of gene enrichment related to ASD (Figure 7(a)) and related to EP (Figure 7(b)). We also extracted the pathways where the *P*value < 0.05 from the KEGG analysis of the genes related to ASD (Figure 7(c)) and EP (Figure 7(d)). The results were indicated that both T cell-related immune response and lipid metabolism had a bearing on two diseases in both biological process analysis and KEGG analysis.

### 3.4. Differential Gene Analysis in ASD and EP

**Figure 8:**
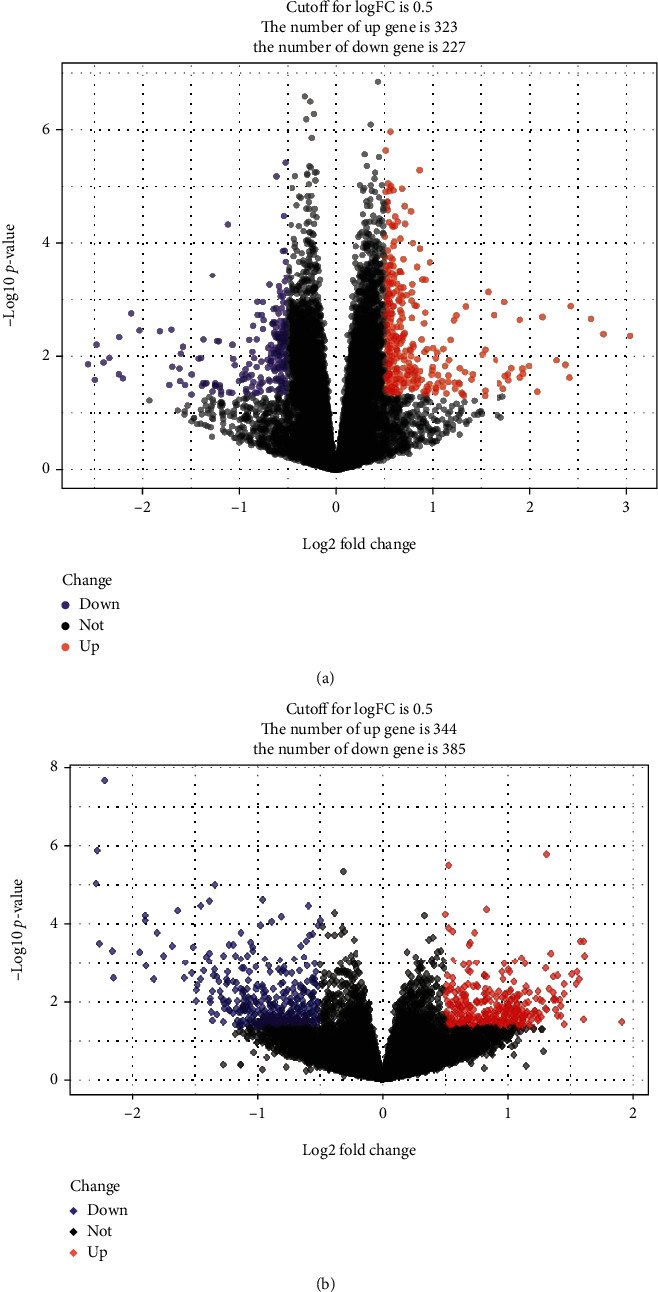
(a) Volcano map of ASD differentially expressed genes. (b) Volcano map of EP differentially expressed genes.

**Figure 9:**
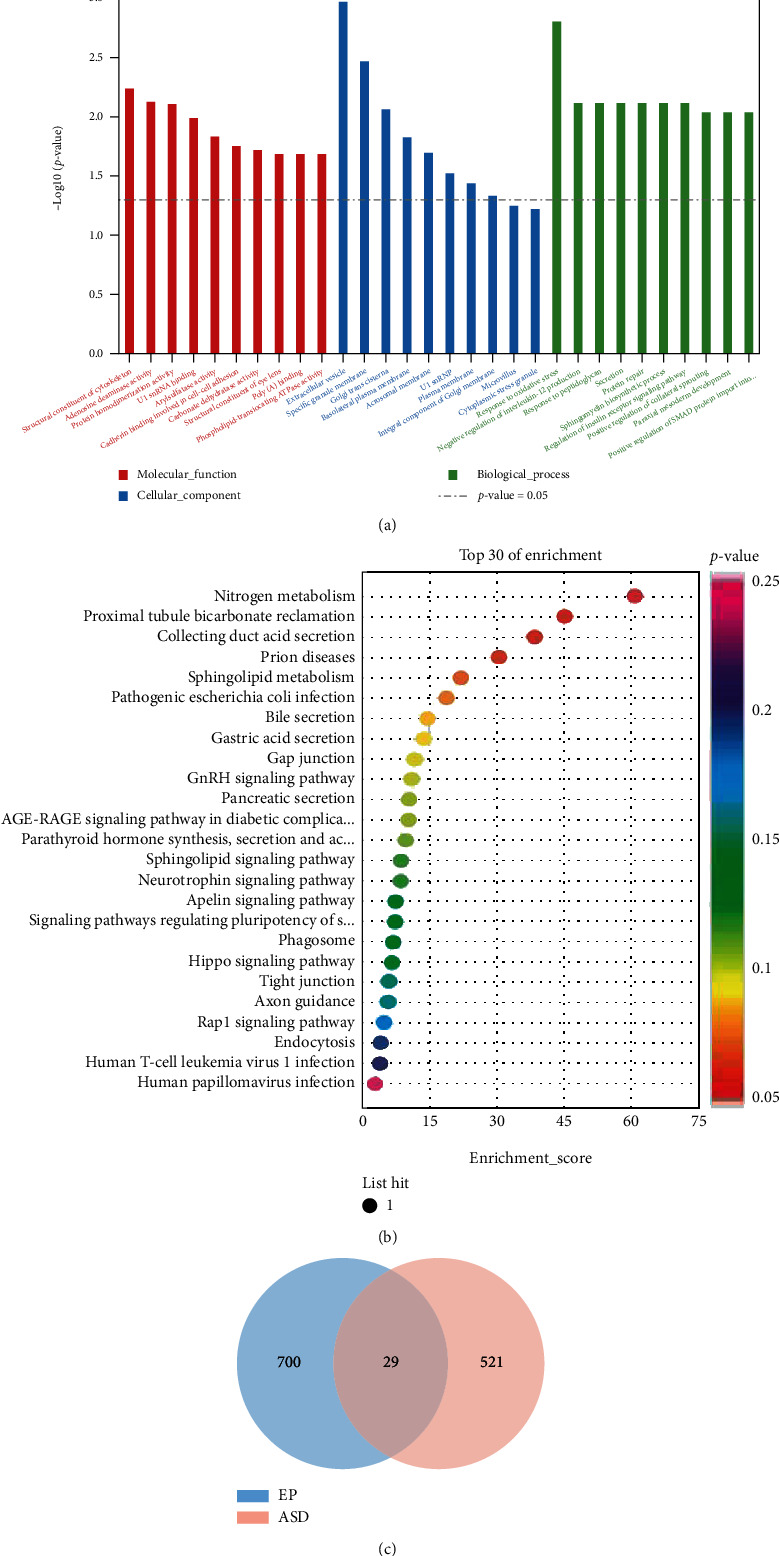
The functions regulated by crosstalk genes of ASD and EP. (a) GO analysis of the crosstalk genes. (b) KEGG analysis of the crosstalk genes. (c) Venn diagram of differentially expressed genes of ASD and EP.

We performed the differential gene analysis on the GSE18123 and GSE7486 datasets. By setting the cut-off value as *P* < 0.05 and ∣log2FC | >0.5, a total of 550 DEGs, we included 233 upregulated and 227 downregulated genes that were identified in the comparison between the peripheral serum of ASD patients and the control samples (Figure 8). For EP, a total of 729 DEGs, including 344 upregulated and 385 downregulated genes, were identified. There were 29 genes (Figure 9(c)) overlapped in the DEGs of ASD and EP. The GO terms of gene enrichment were shown in Figure 9(a), and the pathways of gene enrichment based on KEGG were shown in Figure 9(b). Although these genes were not enriched into immune-related GO terms or KEGG pathways, they were associated with sphingolipid metabolism. The *P* value of sphingomyelin biosynthetic process is 0.00747, and the *P* value of glycosphingolipid metabolic process is 0.0426, which belongs to biological process. For KEGG analysis, the *P* value of sphingolipid metabolism is 0.0445.

### 3.5. The Role of Sphingolipid Metabolism in ADHD

**Figure 10:**
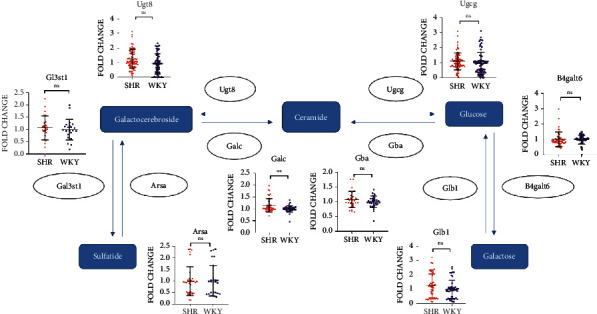
Fold change expression of the objective genes in SHRs and WKYs based on GEO database.

**Figure 11:**
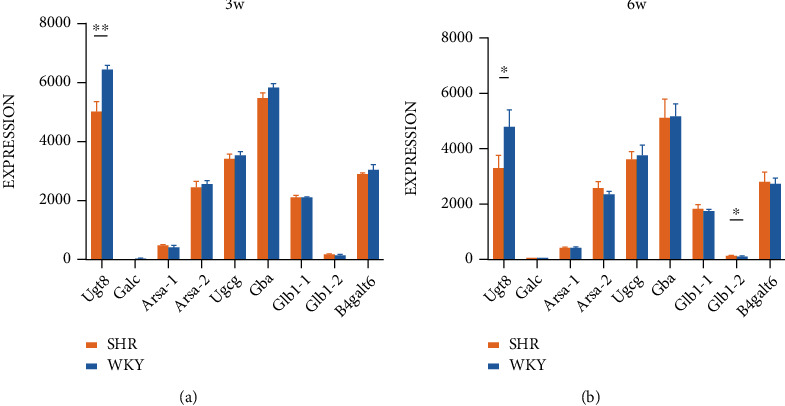
Expression of the objective genes in whole brain of three-week-old and six-week-old model rats of ADHD.

**Figure 12:**
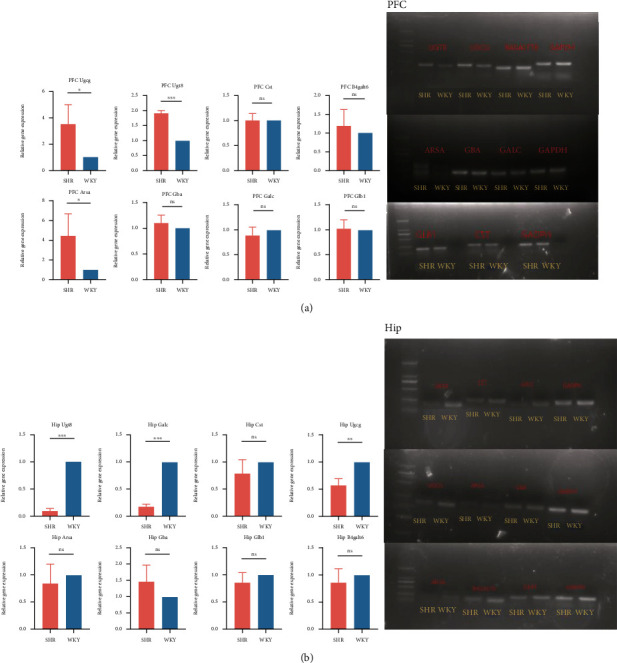
Expression of the objective genes in specific brain regions detected by QPCR and PCR. (a) Expression of the objective genes in PFC of model rats of ADHD. (b) Expression of the objective genes in Hip of model rats of ADHD.

The combination of WGCNA, the differential gene analysis, and the analysis of DisGeNET database showed that lipid metabolism, especially sphingolipid metabolism, played an important role in the ASD and EP. According to our previous research, CycloManN propanyl perac (CycloManN pro), an oligosaccharide compound similar to structure of sulfatide, could promote the growth of neurites of PC12 cells and played a certain role in nerve differentiation [16]. It prompted that the imbalance of sphingolipid metabolism might not only affect the occurrence and development of ASD and EP but also affected the pathophysiological process of other nervous system diseases. With Wistar Kyoto rats (WKYs) regarded as a control strain, spontaneously hypertensive rats (SHRs) are considered as a validated animal model of ADHD [17]. To verify our assumption, the expression of genes associated with sulfatide metabolism in ADHD model rats based on GEO database was analyzed at first. We counted the fold change expression of the objective genes, including Ugt8, Ugcg, Gba, Galc, Gal3st1, Glb1, Arsa, and B4galt6, from GSE1675, GSE2116, GSE8051, GSE8796, GSE12457, GSE41552, GSE53363, and GSE144548. We found that the expression of Galc was significantly upregulated in SHRs. In addition, the expression of Ugt8 also had an upregulated trend in SHRs (Figure 10). To investigate the differences in the expression of lipids in different tissue, we analyzed the expression of these genes in brain tissues of SHRs and WKYs individually. The results suggested a significant difference in the expression of Ugt8 and Glb1 in whole brain tissue (Figure 11). In addition to the analysis of GEO database, we also detected differential expression of the objective genes in PFC and Hip of SHRs and WKYs. The behavior of SHRs and WKYs was evaluated first; then, the general activity level and anxiety level of SHRs were evaluated by open field test, and their activity level and nonselective attention were evaluated by Làt maze. We found that SHRs had significantly more square crossing and rearing than WKYs (Supplementary Figure S1), which showed that SHRs had significantly increased activity and vulnerable attention, which was consistent with the symptoms of ADHD. The expression of the objective genes was detected. As shown in Figure 12, the expression of Ugcg and Ugt8 was significantly upregulated in PFC of SHR rats compared to WKY rats. In addition, the expression of Ugt8 and Galc was downregulated, and the expression of Ugcg was upregulated in Hip.

## 4. Discussion

ASD is a common neurodevelopmental disorder in children with a prevalence of approximately 6.2% [1]. Even though it has a significant genetic component, little is known about how hereditary predisposition leads to its emergence [18]. More than 70 million people worldwide suffer from EP, with an average of 7.6 per 1,000 people living with EP throughout their lives [19]. Similarly, EP has a strong genetic predisposition [18]. The frequent comorbidity phenomenon of ASD and EP suggests that they could share a common genetic basis. Therefore, identifying the shared genetic variations involved in both diseases can help further understand the development of ASD and EP. We used publicly accessible databases to identify and screen crosstalk genes and related pathways in ASD and EP through various bioinformatic methods. The genetic link between ASD and EP was explored by using WGCNA. Based on WGCNA, we found that abnormal immune response and lipid metabolism pathways are enriched in ASD and EP. Consequently, the abnormality of immune system might be a key to the common neurophysiological mechanism between ASD and EP. However, the data from DisGeNET database and other datasets in GEO database only supported the lipid metabolism, instead of immune response, as the important player in ASD and EP development. With the participation of different analysis, we uncovered the sphingolipid metabolic pathway, and it was the key biological pathways causing the coincidence of ASD and EP. Previous studies have found abnormal development in myelination in both ASD and EP [20–25], but little has been associated with their comorbidity. The results of our study suggest that abnormal sphingolipid metabolic pathway affects the development of ASD and EP by modifying myelination. Interestingly, the result of a previous experiment conducted by our group identified an oligosaccharide compound that shared a similar structure with sulfatide, which involves in the sphingolipid metabolic pathway [16]. This compound can promote the growth of neurites of PC12 cells and play a certain role in nerve differentiation. Consequently, we suspected that the abnormal sphingolipid metabolic pathway may cause pathogenesis of comorbidities in other neurological disorders in addition to ASD and EP. It has been reported that abnormal myelin formation may be an underlying pathophysiological process of ADHD [26, 27], but the association of sulfatide-related metabolic pathway with ADHD is unknown. We analyzed the expression of the genes related to sulfatide metabolism in ADHD animal model based on GEO database and found that there were significant differences in involved gene expression. Moreover, we detected the expression of these genes in specific brain regions, and there are still expression abnormalities. Our hypothesis was confirmed that there were abnormal expressions of sulfatide-related metabolic genes in ADHD that may contribute to the development of ADHD by affecting myelination. Our study suggests that sphingolipid metabolism is not only involved in ASD and EP but also ADHD, which is not correlated with those two diseases. This result implies that the sphingolipid metabolism pathways are possibly involved in multiple other neurological disorders. Further investigations, possibly involve clinical samples, are necessary to find a specific sphingolipid that is involved in ASD, EP, and other neurological diseases. This sphingolipid can be a potential drug target that might deter the development of these neurological diseases. As heterogeneous diseases, the clinical characteristics of ASD, EP, and ADHD are different, and the diseases are affected by many factors. The analysis based on large-scale data will undoubtedly obtain more detailed and accurate results. However, due to the lack of relevant databases at present, we only screened out four data sets that met the selection criteria for blood sample sources and thus to reach a conclusion based on small samples first. The mechanism of abnormal sphingolipid metabolism pathway in the pathogenesis of neurological diseases still needs to be further verified by large-scale data sets, especially brain tissue data and model animal experiments.

## 5. Conclusion

We identified the crosstalk genes between ASD and EP based on WGCNA and found abnormal lipid metabolism, especially the sphingolipid metabolism, playing an important role in comorbidity of ASD and EP. Moreover, we found that there was abnormal gene expression of sphingolipid metabolism in ADHD animal model by GEO database analysis and molecular experiment. In summary, abnormalities in the sphingolipid metabolism are involved in the development of ASD, EP, and ADHD, which may affect many other neurological diseases by regulating the process of myelination.

## Figures and Tables

**Table 1:** The detailed information of included datasets.

ID	Series	Platform	Disease	Case	Control	Sample type
1	GSE42133	GPL10558	ASD	91	56	Blood
2	GSE143272	GPL10558	EP	34	51	Blood
3	GSE18123	GPL570	ASD	33	66	Blood
4	GSE7486	GPL570	EP	13	17	Blood

**Table 2:** Detailed information for specific primers.

Symbol	Primers
Gba	Sense 5-GCAGCCAGAAGAGAAGTT-3
	Antisense 5-GTGTCAGCATAGGTGTAGAT-3
Ugcg	Sense 5-ATAGCGGAATACGAGTCATT-3
	Antisense 5-GGTCACATTGGCAGAGATA-3
B4galt6	Sense 5-GTTCACTACTCTGGATACAATG-3
	Antisense 5-GGTCACATTGGCAGAGATA-3
Glb1	Sense 5-AACGACACTTCCTCAAGATT-3
	Antisense 5-GGAGTTGCCATAGTTCACA-3
Galc	Sense 5-TGGAAGGTGGTTGATGTTATAG-3
	Antisense 5-ATTGTGGCGGTCATATTGC-3
Arsa	Sense 5-TCTATGTGCCTGTGTCTCTG-3
	Antisense 5-CCTACTCCAAGATGCCACTT-3
Ugt8	Sense 5-GTGGAGTGCTGTTGGAATA-3
	Antisense 5-CTGGAGGCTGTAGTGATTAG-3
Gal3st1	Sense 5-CTGGATGTGCGTCTCTAC-3
	Antisense 5-TGCTCTTCTTGAGGTTGTAA-3
Gapdh	Sense 5-GTATCGGACGCCTGGTTAC-3
	Antisense 5-GCTCCTGGAAGATGGTGATG-3

## Data Availability

The data used to support the findings of this study are available from the corresponding authors upon request.

## Supplementary Materials

Supplementary 1 Figure S1: behavioral experiment of SHRs and WKYs.

Supplementary 2 Table S1: significant modules and genes in ASD and EP based on WGCNA.

Supplementary 3 Table S2: a total of 166 crosstalk genes between ASD and EP.

Supplementary 4 Table S3: GO analysis of 166 crosstalk genes network.

Supplementary 5 Table S4: KEGG analysis of 166 crosstalk genes.

Supplementary 6 Table S5: ASD- and EP-related genes from DisGeNET database.

Supplementary 7 Table S6: the crosstalk genes between ASD and EP based on DEGs.
