# Supplementary figures and images for: Identification of the Shared Gene Signatures between Autism Spectrum Disorder and Epilepsy via Bioinformatic Analysis
Source: Comput Math Methods Med. 2022 Dec 16;2022:9883537. doi: 10.1155/2022/9883537 (PMC9806688; doi:10.1155/2022/9883537)

Supplementary 1. Figure S1 Behavioral experiment of SHRs and WKYs.


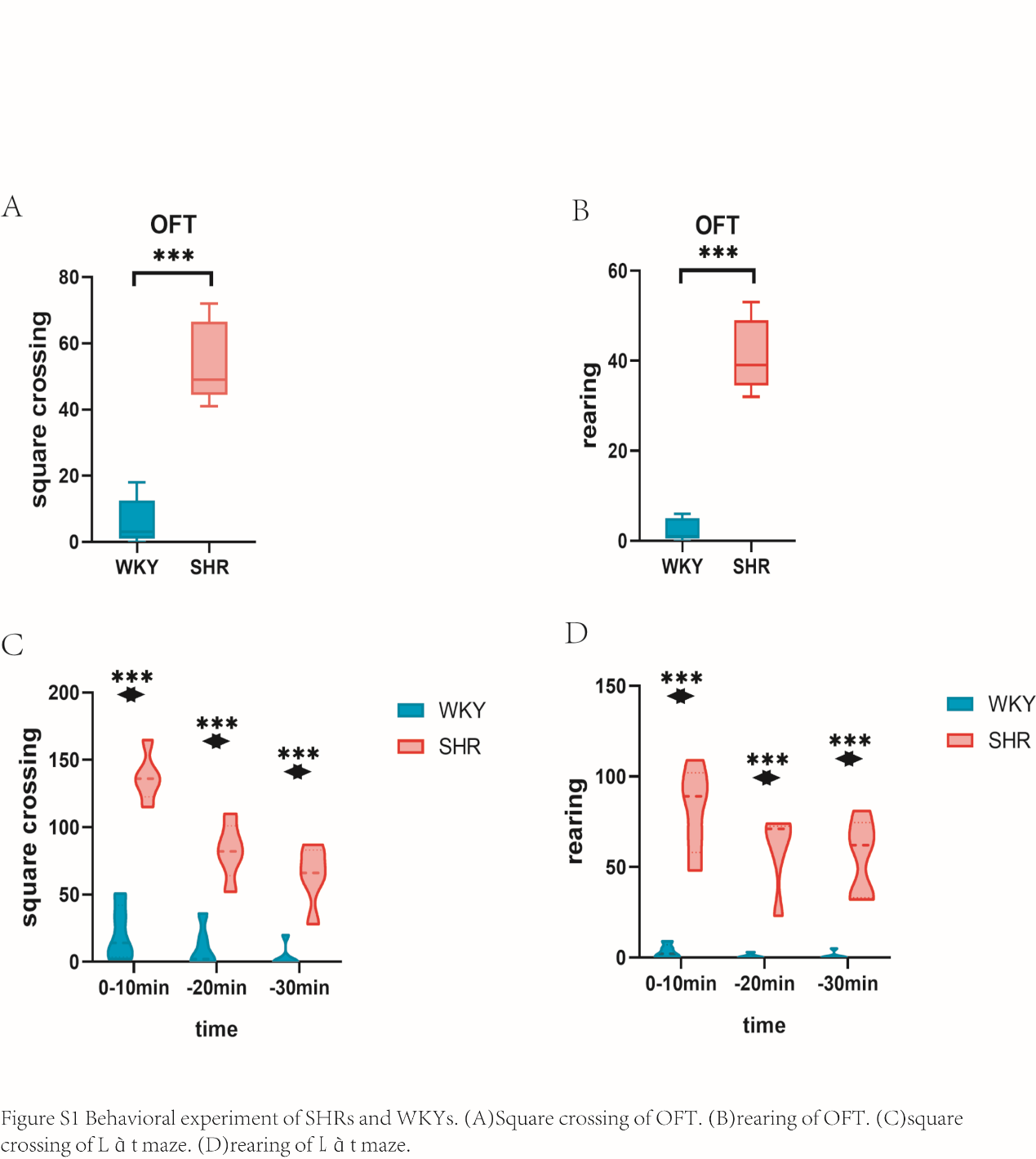

Supplement: Supplementary 1 — Figure S1: behavioral experiment of SHRs and WKYs. [file 9883537.f1.docx]
